# Supplementary material for: Are carbon emissions trading and green financial instruments synergistic? -Comprehensive quantitative research based on content analysis
Source: PLoS One. 2024 Mar 7;19(3):e0298601. doi: 10.1371/journal.pone.0298601 (PMC10919601; doi:10.1371/journal.pone.0298601)
Supplement: S1 Data — (ZIP) [file pone.0298601.s001.zip › Data/地级市Z_F工作报告.zip!/╣π╢1⁄2/║╙╘┤╩╨/└┤╫╘╣1⁄2╓┌║┼í╛┬φ┐╦╩2╛▌═°í┐_2011.docx]

各位代表：

现在，我代表市人民政府向大会作政府工作报告，请予审议，并请各位政协委员和其他列席人员提出意见。

2010年工作回顾及“十一五”重大成就

2010年，是实施“十一五”规划的最后一年，也是抓落实、打硬仗、出战果的决胜之年。市政府在省委、省政府和市委的正确领导下，在市人大及其常委会和市政协的监督支持下，以科学发展观统揽全局，全面贯彻落实党中央、国务院和省委、省政府一系列决策部署，按照市委五届七次、八次全会的要求，立足增总量、优结构、扩需求、重民生、强后劲，以承办世界客属第23届恳亲大会为契机，攻坚克难，奋力冲刺，经过全市人民的共同努力，打赢了一系列事关河源发展全局的大仗硬仗，取得了一系列重大战果，依期完成了市五届人大五次会议确定的各项目标任务和“十一五”规划目标。

——国民经济加快发展，综合实力再上新台阶。面对国际金融危机冲击持续影响、国内金融政策调整、节能降耗压力加大等突出困难和挑战，我们始终把做大做优经济“蛋糕”作为头等大事，全力以赴推动全市经济社会稳健较快发展。预计全市完成生产总值474.6亿元，比上年增长13.1%，增速比上年加快3个百分点，其中第一产业增加值61.9亿元，增长6.1%；第二产业增加值262.1亿元，增长19.1%；第三产业增加值150.6亿元，增长6.4%；三大产业结构由上年的13.1∶52∶34.9调整为13∶55.2∶31.8。人均生产总值15985元，增长10.9%。全社会工业总产值和增加值分别为883亿元和230亿元，分别增长24.8%和21.9%，总量排全省第15位。地方财政一般预算收入25.1亿元，增长24.3%，提前一年实现翻一番目标；全社会固定资产投资242.7亿元，增长22.5%；实际利用外资1.68亿美元，总量居全省第14位；外贸出口17亿美元，增长20 %，总量居山区市之首；全社会消费品零售总额163.1亿元，增长17.2%。民营经济快速发展，民营经济增加值占全市生产总值比重达53.8%。富县强镇战略取得初步成效，县域生产总值、工业增加值、地方财政一般预算收入分别增长11%、18%、19%，紫金、东源和龙川3县财政综合增长率在全省排名分别前移19、12和5位。

——发展方式加快转变，产业升级实现新突破。我们站在确保经济社会可持续发展和有效增强产业竞争力的战略高度，积极践行“三反”理念，加快推进产业转型升级和建立现代产业体系。“四新”产业加速集聚。围绕打造环珠三角新兴产业集聚地，以中山（河源）产业转移园和各县区工业园区为主要载体，加大招商选资力度，大力培育发展新电子、新能源、新材料、新医药等战略性新兴产业。市高新区实现工业总产值200.3亿元，税收5.7亿元，分别增长62%和17.6%；以手机为主的电子信息产业、以模具和硬质合金为主的新材料产业及以太阳能电池为主的光伏产业等主导产业进一步形成，其中手机产量达到3000万台，比上年翻了一番；继引进总投资200亿元的汉能太阳能薄膜电池生产项目后，又成功引进了总投资100亿元的中兴通讯研发生产项目、首期投资60亿元的恒泰豪太阳能光伏项目，在2010年全省省级产业转移园考核中获总分第一名，并被评为“全国十大最具发展潜力开发区”。各县区工业园建设加快，罗湖（源城）产业转移园竞得省1亿元扶持资金，东源县引进了旗滨、南玻光伏玻璃生产项目。万绿湖直饮水项目前期工作扎实推进。在“四新”产业的带动下，全市工业经济持续快速发展，实现规模以上工业总产值 838亿元，增长26.2%；增加值215亿元，增长23.8％。其中“一区六园”完成工业总产值501亿元、工业增加值134亿元，分别增长28%和27.2%。生态旅游蓬勃发展。“客家古邑、万绿河源、温泉之都、恐龙故乡、红色经典”五大旅游品牌进一步打响，全年接待入市游客达1050万人次，旅游总收入45.5亿元，分别增长25%和27%。《万绿生态旅游度假区策划》通过专家评审。投入2亿多元对万绿湖、镜花缘、苏家围等主要景区景点改造升级。和平温泉之都度假区被评为国家4A级旅游景区。引进总投资超过100亿元的东江·巴登城、东江源温泉度假村、广晟御临门温泉二期等一批大型高端旅游产业项目。投入20多亿元新建改造了一批星级酒店，万绿湖东方国际酒店建成营业，希尔顿大酒店加快建设，市区三星级以上酒店床位从2000多张增加到6000多张，成为全省山区旅游接待能力最强的地区之一。现代服务业茁壮成长。围绕建设粤北赣南区域物流中心，首期投资65亿元的东江商贸物流城加快建设，总投资30亿元的市商业中心正式动工，占地35万平方米的市大型农副产品批发中心前期工作加快推进。供销新网工程建设进展顺利。房地产、金融保险、商贸流通、中介信息等其他服务业加快发展。科技创新能力不断提高。积极推进企业自主创新，建成市级科技企业孵化器、企业创新服务中心等一批公共科技服务平台，创建了4家省级工程技术研究开发中心，扎实推进省产学研结合示范市创建工作。

——环境保护力度加大，宜居城乡建设跃上新水平。在刚性支出显著增加、财政收支矛盾十分突出的情况下，多渠道筹集资金30多亿元，高标准建成了一批城乡重大基础设施和生态环保设施，宜居城乡建设取得了重大进展。城镇化率达42.4%，提高1.8个百分点；市区交通建设实现重大突破，城区框架面积成倍扩大；创建国家卫生城市通过了国家技术评估，创建国家环保模范城市各项指标全面达到国家考核要求，创建全国双拥模范城市取得显著成效。城乡规划进一步完善。《河源市城乡总体规划》通过省专家评审，《河源市土地利用总体规划》、《河源市公园城市规划》等专项规划编制完成，市区近期建设用地控制性详细规划实现全覆盖。交通建设力度空前。总投资24亿元的河源迎客大道、万绿湖大道、滨江大道（一期）、西环路（一期）、东江西路、珠河大桥新桥等市区“五路一桥”建成通车，市区面貌焕然一新。大广高速公路连平段进入征地阶段，河紫、汕昆、粤湘、东环等高速公路前期工作顺利推进。大环市路建设加快推进，龙紫公路基本建成。完成国省道大中修136公里、县道路面大修150公里、村道硬底化573公里。电力电网建设扎实推进。河源电厂一期工程全面建成运营，农村电网改造年度计划全面完成，建成“1·8·28”输变电工程项目13个，建成110千伏以上输电线路606千米。环境保护和生态建设成效显著。河源发展低碳经济的经验在墨西哥坎昆联合国气候变化大会上推介。9座日处理能力22.5万吨的污水处理厂全面运营，建成七寨生活垃圾卫生填埋场，正在建设7座日处理能力23.7万吨的污水处理厂。严格实行节能减排问责制，万元GDP能耗下降0.5%。林业体制改革全面完成并在全国会议介绍经验，集体林权制度主体改革任务基本完成，全面实施封山育林工作，火灾案数和受害面积分别下降89.2%和95.7%，森林覆盖率达71.2%，继续居全省第一。市区客家文化公园动工建设，完成笔架山公园、龟峰公园提升改造，建成梧桐山公园、文化广场体育休闲新区和15个小公园，市区人均公共绿地面积达12平方米，建成区绿化覆盖率达44.1%。完成“万村绿”工程示范村建设83个，绿化村道194公里。顺利完成矿产资源整合任务并通过省政府验收考核。城市管理水平明显提高。进一步完善城市管理体制，组建城市管理综合执法队伍，加大监管力度，有力遏制城市“六乱”现象。市区农贸市场建设和升级改造年度计划顺利实施；组建新的公交运营公司，全面更新了公交车辆，新开通市区线路10条、近郊线路2条。对“五小”行业和中小餐饮业开展了专项整治。加强了城中村和城乡接合部整治。宜居县城建设步伐加快，龙川、和平县城及部分中心镇的建设出现新亮点。

——新农村建设扎实推进，脱贫奔康取得新成果。全面落实强农惠农政策。全市共发放种粮、家电和汽车下乡、以旧换新等政策补贴资金2.7亿元，比上年增加80%。扶贫“双到”成效显著。各级各部门为省认定的贫困村投入扶持资金2.75亿元，启动建设21个农业基地，引导1.3万人到转移园就业，农房改造竣工户数9814户，超额完成年初目标。扶贫“双到”工作被省评为优秀等次。全面落实移民后扶政策，成功争取省支持建设东源水库移民“双转移”示范基地。“千村脱困”政策全面落实，1117个贫困村全年共获分红款2601万元。农村经济稳步增长。全市农林牧渔业实现总产值100亿元，增长6.5%。东江上游特色水果产业带项目建设步伐加快。编制了灯塔盆地现代生态农业发展规划。新增省级现代农业园区3个、现代农业示范区2个，有8个企业入选广东省现代农业项目100强。“菜篮子”工程建设得到加强。龙川县被评为全国特色农业加工基地。农村面貌不断改善。新增新农村建设示范点102个，编制了《河源市新农村建设农房改造设计样板》。新建硬底化村道247公里，改厕2.3万户，对旅游景区所在行政村优先实施“农村清洁工程”。水利设施进一步完善。总投资12.6亿元的7宗城乡水利防灾减灾工程基本完工，新建加固防洪堤199.4公里，连续五年获评省一等奖，市区防灾减灾工程为全省示范样板工程。建成农村饮水安全工程9宗，解决了农村8.5万人饮水不安全问题。中小型水库除险加固和中小河流治理工程如期推进。

——财税金融快速增长，保障水平实现新提高。财税总量跃上新台阶。在政策性减收因素增多等大背景下，全市财政一般预算总收入达到104亿元，其中地方财政一般预算收入增速超过预期目标11.3个百分点。完成总税收52亿元，增长27.2%，增速提高18.4个百分点。金融机构各项存款余额500亿元，增长22.1%；贷款余额338.7亿元，增长15.1%；存贷比为67.7%,居全省第2位;城乡居民存款余额314.3亿元，增长19.2%。财税管理水平不断提升。市、区的财政管理体制进一步完善，紫金县纳入省直管县试点。部门预算改革不断深化，国库集中收付改革稳步推进，财政资金监管进一步规范。财政质量进一步提高，非税收入比重下降至18.9%，税收占财政比重居全省第七位。国有资产实现保值增值。由我市实践和总结的“新时期审计文化”在全国推广。保重点保运转保增长成效显著。积极调整和优化支出结构，实现财政收支平衡。有效筹集资金，确保了市区“五路一桥”等重大项目如期建成。足额安排配套资金2.15亿元，推动扩大内需项目加快建设。成功争取上级扶持项目127个，扶持资金达7.43亿元。在市本级财力仍然薄弱的情况下，支持县区资金3.34亿元。帮助企业争取上级扶持资金1亿元，争取金融机构为政府重大项目签约融资27.1亿元，新增放贷到位13.5亿元；为中小企业融资113.8亿元，增长43.8%；为“三农”项目融资76.4亿元，增长77.5%。争取省新批准成立3家小额贷款公司，实现县区服务全覆盖目标；成立了全市首家农村商业银行，并顺利完成首家村镇银行组建工作。

——教育事业加快发展，文化建设展现新气象。启动了创建教育强市和建设文化河源工作，形成了以“胸怀理想、艰苦奋斗、务实创新、众志成城”为主要内容的新时期河源人精神，河源的客家历史文化地位显著提升。教育事业成就突出。高中阶段教育毛入学率提高12.8个百分点，提前一年实现“普高”目标。市理工学校二期工程竣工并通过国家级重点职校评估验收，市技工学校通过高级技工学校评审，启动了市卫生学校新校区征地拆迁和市技工学校图书馆建设工作。义务教育质量不断提高，小学、初中学龄人口入学率分别达99.96%和99.6%，初中毕业生升学率达91.9%。全市改造中小学校舍160所，市区扩建了河源中学、市田家炳实验中学，新建金沟湾小学、雅居乐学校等一批小学。完成了中小学教师“代转公”工作。市委党校教研大楼建成投入使用。文化建设喜获丰收。承办第23届世客会取得圆满成功，受到海内外客属乡亲的高度赞誉。建成市职工文化活动中心、恐龙博物馆。建立一批中共党史和革命传统教育示范基地。编辑出版了《客家古邑文化》丛书，举办了国际客家学术研讨会，创办了全球第一部客家电子杂志《古邑客家》，《天下客家》获中国南派优秀纪录片奖和最佳摄影奖。社会科学理论研究不断加强。开展文物普查，推进文化旧（遗）址保护修缮，完成了佗城、林寨古村落保护改造首期工程和阮啸仙故居修缮。源城区成功创建“南粤锦绣工程”文化先进区。忠信花灯被列入全国非物质文化遗产名录。新建农村文化室264个，城乡群众文化活动更加丰富多彩。文化产业发展步伐加快，文化体制改革扎实推进。

——社会事业全面进步，改善民生取得新成效。财政安排民生支出达68.34亿元，增长26%，占一般预算支出的73.6%。筹集资金37.1亿元，全面完成“十件实事”和“十项惠民工程”年度任务，在公共交通、公园建设、市场改造、教育事业、医疗卫生、社会保障等方面为群众解决了一大批热点难点问题，群众对各级政府的满意度进一步提升。人民生活持续改善。城镇居民人均可支配收入13177元，农村居民人均纯收入5530元，分别增长8.6%、10.3%。保障水平明显提高。全市参加企业职工基本养老、失业、医疗、工伤、生育保险人数分别增长5 %、5 %、9 %、5 %、19 %，并实现了五个险种市级统筹；农村合作医疗参保率达98.2%，提高2.8个百分点；新农保试点扎实推进，低保覆盖面不断扩大，困难群众实现应保尽保。政策性农村住房保险承保率达100%，远超全省20%的承保水平。新增经济适用住房和廉租住房1100多套，为170多户单亲特困母亲建安居房提供配套资金。扎实做好就业再就业工作，农村劳动力转移增加7.1万人次，全市转移园吸纳本市劳动力10.7万人，年末城镇登记失业率2.9%。社会事业协调发展。建成市中医院新院、市职业病和慢性病防治院，启动了市精神卫生防治中心建设；投入3亿多元全面推进医疗卫生体制改革，全面提高基本医疗保障水平，近三分之二乡镇卫生院实施国家基本药物制度。第六次全国人口普查进展顺利。全面完成人口计划指标，稳定了低生育水平，“两无”覆盖率大幅提升，计生后进地区转化成果进一步巩固。源城区被评为国家级计划生育优质服务先进单位。全民健身运动深入开展，新建行政村标准篮球场180个，参加第16届亚运会和第13届省运会获得可喜成绩。与马来西亚亚庇市建立友好城市。人防指挥体系不断健全。社会保持和谐稳定。“平安亚运、和谐世客”目标圆满完成，公众安全感和人民群众对公安工作满意度显著提升。县、镇、村三级综治信访维稳平台建成使用。人民调解、法制宣教和法律援助工作有效推进。安全生产形势稳定好转，生产安全事故起数下降27.6%，未发生重特大安全事故。应急管理体系进一步健全，处置能力不断提高。一批涉法涉诉、重点信访和老信访问题得到依法妥善处理，信访和越级上访人数和批次减少。连平县被评为全国法治县创建先进单位。法制、民族宗教、机关事务、行政服务、接待、驻外机构、知识产权、科协、物价、信息、对台、打私、档案、史志、气象、妇女、儿童、老龄、残疾人和民兵预备役等各项工作取得新成绩。

——政府建设不断加强，执政能力实现新提升。把抓落实、打硬仗、出战果作为政府作风建设的主题，进一步加大问责督办力度，强化“网格化”和层级管理。特别是在时间紧、任务重、要求高的情况下，全力破解资金筹集、征地拆迁、项目施工等难题，如期完成了迎世客系列项目建设，为世客会的顺利召开创造了重要条件。认真落实党风廉政建设责任制和领导干部廉洁自律各项规定，加强各级政府领导班子和公务员队伍作风建设，进一步树立团结拼搏、务实高效、依法行政、廉洁从政的良好形象。主动接受人大及其常委会的依法监督、人民政协的民主监督和社会各方面的监督，注意听取各民主党派、工商联和无党派人士的意见和建议。全年办理市人大代表建议99件、市政协委员提案100件，代表和委员满意率分别达97.8%和100%。

各位代表，2010年各项工作任务的完成和预期目标的实现，标志着我市成功应对了异常困难的局面，经受住了空前严峻的考验，全面超额完成了我市“十一五”规划各项目标任务，经济社会发展又迈上了一个新台阶，使河源进入了一个跨越发展、加快崛起的新阶段，也为“十二五”发展奠定了更为坚实的基础。回顾“十一五”，我们深切感到：

“十一五”时期的五年，是我市综合实力显著提升的五年。五年间，全市GDP翻了一番多，年均增长16.6%，增速排全省第4位；人均GDP翻了一番，年均增长14.7%；地方财政一般预算收入增长1.95倍，年均增长24.1%，超过规划目标4.1个百分点；税收总收入增长1.9倍，年均增长23.6%；全社会工业增加值接近翻两番，年均增长28.6%，超过规划目标6.6个百分点；全社会工业总产值翻了两番，年均增长28.8%。三大产业结构由2005年的20.7∶39.3∶40调整为13∶55.2∶31.8。县域财力大幅提升，其中源城区、东源县、连平3县地方财政一般预算收入突破3亿元。全市主要经济指标绝对值在全省21个地级以上市排名全面前移，其中全市生产总值、规模以上工业增加值、地方财政一般预算收入分别比2005年前移1位、3位和1位；城市综合增长竞争力2008年和2009年在全国地级城市分别排第二位和并列第一位，成为环珠三角地区发展速度最快、发展质量最好、发展后劲最足的地区之一。

“十一五”时期的五年，是我市城乡面貌显著变化的五年。五年来，全市城镇化率提高10个百分点，市区中心城区规划面积从50平方公里扩大到250平方公里，建成区面积从25平方公里扩大到39平方公里；成功争取一批高速公路提前规划实施；改造建设市区重点道路30.8公里，构建了更为方便快捷的城市道路网络；全市生态环境质量进一步巩固提升，市区人均公共绿地面积和建成区绿化覆盖率分别提高52.8%和16.3%，东江等主要河流水体水质继续保持国家地表水Ⅰ—Ⅱ类标准，空气质量常年保持国家一级水平，成为全省唯一无酸雨的地级市，连续7年在全省环境质量考核和珠江综合整治考核中获得“双优秀”，全面完成节能减排目标，先后被评为“全国生态环境保护最佳范例城市”、“中国节能减排二十佳城市”、“中国十大特色休闲城市”、“中国绿色经济十佳城市”。

“十一五”时期的五年，是我市人民生活显著改善的五年。五年来，各级政府把越来越多的财力用于改善人民生活，涉及交通设施、文化教育、医疗卫生、社会保障等领域，其中2010年地方财政一般预算中民生支出比2005年增加2.2倍，年均增长26.4%。特别是2007年以来，累计投入89.9亿元，积极实施以改善民生为主要内容的“十项惠民工程”和“十件实事”，有力推动了社会各项事业发展和人民生活水平提高。对比“十五”期末，城乡居民储蓄存款余额翻了一番多，城镇居民人均可支配收入和农村居民人均纯收入分别提高55.2%和46.9%；省认定贫困村贫困户和贫困人口分别下降41.3%和41.7%。

各位代表，五年的发展成就令人鼓舞，五年的实践经验弥足珍贵。这就是：必须始终坚持加快发展不动摇，深刻认识河源的基本市情，着力做大做优经济“蛋糕”特别是壮大工业经济总量，切实提高产业竞争力，才能从根本上改变河源相对落后的状况；必须始终坚持可持续发展不动摇，注重打基础、谋长远，不断巩固提升生态优势，加快转变发展方式，才能实现经济发展与环境保护的“双赢”；必须始终坚持抓落实、打硬仗、出战果不动摇，面对困难不退缩，碰到矛盾不回避，只争朝夕不拖拉，不断提高各级政府的执行力，才能有效破解发展瓶颈，确保各项工作干一件成一件；必须始终坚持执政为民不动摇，实实在在为老百姓办实事谋福祉，不断提升老百姓幸福感，才能赢得人民群众的充分理解、信赖、拥护和支持；必须始终坚持依法行政不动摇，从严治政，廉洁从政，规范办事，对事关河源核心利益和发展大局的事情敢抓善管，才能不断提高依法科学民主决策水平和管理社会的能力；必须始终坚持团结干事不动摇，调动一切积极因素，营造和谐发展环境，凝聚各方发展力量，才能形成更强大的工作合力。

过去的五年，成绩来之不易。这是省委、省政府和市委正确领导的结果；是市人大、市政协和社会各界监督支持的结果；是全市人民抢抓机遇，在竞争中迎接挑战、在转型中经受考验、在前进中赢得胜利的结果；是历届市委、市政府班子艰苦创业、打下良好基础的结果；也与深圳、中山等兄弟市和中央驻粤单位、省直单位的大力帮扶以及社会各界的大力支持密不可分。在此，我代表市人民政府，向全市广大干部群众，向各位人大代表和政协委员，长期关心支持我市发展的各级领导和各界朋友，积极参与河源建设的海内外客商和外来务工人员，驻河源部队和武警、消防官兵，各民主党派、工商联、人民团体、社会各界人士，表示最衷心的感谢和最崇高的敬意！

在充分肯定成绩的同时，我们也清醒地看到存在的困难和问题。主要是：经济总量仍然太小，财政收支矛盾仍然突出，县域经济发展不平衡，加快发展和转变经济发展方式的任务仍然十分艰巨；城镇化水平不够高，公共基础设施有待完善，实现基本公共服务均等化任重道远；社会事业发展水平总体滞后，人民生活水平仍然不高，改善民生和环境保护压力巨大；一些干部的观念、水平、作风还不能完全适应新形势发展要求。这些困难和问题，都要在今后工作中采取有力措施加以解决。
